# Antibacterial Activity of Novel Cationic Peptides against Clinical Isolates of Multi-Drug Resistant *Staphylococcus pseudintermedius* from Infected Dogs

**DOI:** 10.1371/journal.pone.0116259

**Published:** 2014-12-31

**Authors:** Mohamed F. Mohamed, G. Kenitra Hammac, Lynn Guptill, Mohamed N. Seleem

**Affiliations:** 1 Department of Comparative Pathobiology, College of Veterinary Medicine, Purdue University, West Lafayette, Indiana, United States of America; 2 Department of Veterinary Clinical Sciences, College of Veterinary Medicine, Purdue University, West Lafayette, Indiana, United States of America; Academia Sinica, Taiwan

## Abstract

*Staphylococcus pseudintermedius* is a major cause of skin and soft tissue infections in companion animals and has zoonotic potential. Additionally, methicillin-resistant *S. pseudintermedius* (MRSP) has emerged with resistance to virtually all classes of antimicrobials. Thus, novel treatment options with new modes of action are required. Here, we investigated the antimicrobial activity of six synthetic short peptides against clinical isolates of methicillin-susceptible and MRSP isolated from infected dogs. All six peptides demonstrated potent anti-staphylococcal activity regardless of existing resistance phenotype. The most effective peptides were RRIKA (with modified C terminus to increase amphipathicity and hydrophobicity) and WR-12 (α-helical peptide consisting exclusively of arginine and tryptophan) with minimum inhibitory concentration_50_ (MIC_50_) of 1 µM and MIC_90_ of 2 µM. RR (short anti-inflammatory peptide) and IK8 “D isoform” demonstrated good antimicrobial activity with MIC_50_ of 4 µM and MIC_90_ of 8 µM. Penetratin and (KFF)_3_K (two cell penetrating peptides) were the least effective with MIC_50_ of 8 µM and MIC_90_ of 16 µM. Killing kinetics revealed a major advantage of peptides over conventional antibiotics, demonstrating potent bactericidal activity within minutes. Studies with propidium iodide and transmission electron microscopy revealed that peptides damaged the bacterial membrane leading to leakage of cytoplasmic contents and consequently, cell death. A potent synergistic increase in the antibacterial effect of the cell penetrating peptide (KFF)_3_K was noticed when combined with other peptides and with antibiotics. In addition, all peptides displayed synergistic interactions when combined together. Furthermore, peptides demonstrated good therapeutic indices with minimal toxicity toward mammalian cells. Resistance to peptides did not evolve after 10 passages of *S. pseudintermedius* at sub-inhibitory concentration. However, the MICs of amikacin and ciprofloxacin increased 32 and 8 fold, respectively; under similar conditions. Taken together, these results support designing of peptide-based therapeutics for combating MRSP infections, particularly for topical application.

## Introduction

Methicillin-susceptible *Staphylococcus pseudintermedius* (MSSP) and methicillin-resistant *S. pseudintermedius* (MRSP) are a leading cause of skin and ear infections and post-operative wound infections in dogs and cats [1], [2]. *S. pseudintermedius* isolates can also cause infections in humans as apparent zoonotic transfer from dogs has been reported [2]–[5]. Similar to methicillin-resistant *Staphylococcus aureus* (MRSA), MRSP is a nosocomial pathogen that can colonize personnel in veterinary hospitals [1], [6]. Recent studies reported that MRSP from Europe and North America emerged resistance to virtually all classes of antimicrobial agents used in veterinary medicine [7]. Such dissemination of multidrug resistant staphylococci among dogs raises concern due to the few therapeutic options available for treatment [8]. Therefore, there is an urgent need for novel antimicrobial compounds with new mechanisms of action.

Antimicrobial peptides (AMPs) serve as an alternative novel therapeutic approach against microbial infections. AMPs constitute the first line of defense against invading pathogens in most multicellular organisms. They have been discovered from a broad range of organisms, from microorganisms to plants and from insects to mammals [9]. AMPs are generally between 12 and 50 amino acids in length with a cationic charge and contains up to 50% hydrophobic amino acids. They have the ability to form an amphipathic secondary structure that allows the peptides to partition into the bacterial membrane lipid bilayer [10].

The mechanism of action of AMPs involves binding to the negatively charged anionic phospholipids on lipopolysaccharide (LPS) of Gram-negative bacteria or to the teichoic acids of Gram-positive bacteria. Once peptides aggregate in sufficient concentration, they destabilize the lipid head groups and produce pores in the cell membrane leading to leakage of cytoplasmic contents and bacterial cell death [9], [11]. However, membrane disruption is not the only proven mechanism of bacterial killing by AMPs. Instead, peptides can traverse bacterial membranes and induce killing through inhibition of specific macromolecular synthesis pathways [11].

Several studies have reported the potency of AMPs in combating *S. aureus* infections [12], [13]; however, to our knowledge, there are limited data about their activity and potential use against *S. pseudintermedius* [14], [15]. In the present study, we investigated the antimicrobial activity of six synthetic short peptides against clinical isolates of MSSP and MRSP from infected dogs. Moreover, we performed a series of experiments to explore their antibacterial mechanism of action. Finally we examined their toxicity toward mammalian cells and assessed the ability of *S. pseudintermedius* to develop resistance to peptides.

## Materials and Methods

### Peptides, antibiotics and reagents

Peptides (RRIKA, RR, WR-12, IK8 “D isoform”, (KFF)_3_K and penetratin) were synthesized by GenScript (Piscataway, NJ) using solid-phase 9-fluorenylmethoxy carbonyl (Fmoc) chemistry and purified to a purity of 98% using reverse-phase high-performance liquid chromatography (HPLC). Peptide mass was confirmed by mass spectrometry (Table 1). Nisin (Sigma, N5764), melittin from honey bee venom (Sigma, M2272), ampicillin sodium salt (IBI Scientific), ciprofloxacin (Sigma), amikacin hydrate (Sigma) and propidium iodide (Molecular Probes, Life Technologies) were all purchased from commercial vendors. Mueller-Hinton broth (MHB) and Mueller-Hinton agar (MHA) were purchased from Sigma-Aldrich, while trypticase soy broth (TSB) and trypticase soy agar (TSA) were purchased from Becton-Dickinson, Cockeysville, Md.

**Table 1:** Amino acid sequence and physicochemical properties of peptides used in this study.

Peptide designation	Amino acid sequence^a^	Length	Molecular weight	Charge	Hydrophobic amino acids
RR	WLRRIKAWLRR	11	1553.9	+5	54%
RRIKA	WLRRIKAWLRRIKA	14	1866.3	+6	57%
WR-12	RWWRWWRRWWRR	12	2072.4	+6	50%
IK8 “D isoform”^1^	irikirik	8	1040.28	+4	50%
(KFF)3K	KFFKFFKFFK	10	1413.7	+4	60%
penetratin	RQIKIWFQNRRMKWKK	16	2246.7	+7	37%

1 D-amino acid substitution.

a Small underlined residues represent D-amino acids.

### Bacterial isolates

Forty isolates of *S. pseudintermedius* (30 MSSP and 10 MRSP) identified at the Indiana Animal Disease Diagnostic laboratory from specimens collected from dogs admitted to the small animal teaching hospital at Purdue University were included in the study (Tables 2 and 3). Clinical specimens were inoculated onto 5% sheep blood agar and incubated at 35°C for 18–24 hours. White, beta-hemolytic colonies with morphology suggestive of *Staphylococcus* sp. were used to inoculate a coagulase tube test and were identified to the species level using the Vitek 2 (BioMérieux). Vitek identification of *S. pseudintermedius* was confirmed by a positive coagulase test. Antimicrobial susceptibility was determined by broth microdilution using the SensiTitre (Trek). Isolates demonstrating resistance to oxacillin, a surrogate for methicillin, with a MIC greater than or equal to 0.5 µg/mL were screened for *mecA* by PCR as previously described [16]. A *mecA* positive result was assigned to samples with a visible 310 bp band on a 1.5% agarose gel.

**Table 2:** Minimum inhibitory concentration (MIC) of peptides against methicillin sensitive *Staphylococcus pseudintermedius* (MSSP) strains.

Isolates	Origin	MIC (µM)						Resistance phenotype
		RRIKA	RR	WR-12	IK8 “D isoform”	Penetratin	(KFF)_3_K	
SP1	urine	1	4	2	4	4	8	PEN, AMP
SP2	urine	2	8	2	8	16	16	PEN, AMP, CLIN, ENRO, ERYTH, GEN,MARBO, TMP-SMX
SP4	urine	2	4	4	8	16	16	PEN, AMP, TMP-SMX
SP6	ear	2	4	1	4	16	8	-
SP7	ear	1	4	2	4	8	8	AMK, PEN,AMP,CLIN,ENR,ERM,GEN,MARB, TMP-SMX
SP10	urine	1	4	1	4	8	8	PEN,AMP,CLIN, ERM, TMP-SMX
SP11	hair	1	4	1	4	8	8	PEN, AMP,CHL,CLIN,
SP12	urine	0.5	0.5	0.5	0.5	8	4	PEN,AMP,AMK,ENR,GEN,MARB, TMP-SMX
SP13	skin abscess	1	4	0.5	4	8	8	PEN,AMP
SP14	ear	2	4	4	4	8	8	PEN,CHL,CLIN,ERM,
SP15	urine	1	4	2	8	8	8	-
SP16	abdominal swab (surgery)	2	8	1	8	8	8	-
SP17	skin abscess	1	8	2	8	32	8	PEN, AMP, AMK,CLIN,ENR,ERM,GEN,MARB, TMP-SMX
SP18	urine	1	4	2	8	8	8	PEN
SP19	synovial fluid(stifle swab)	1	4	1	4	8	8	PEN,AMP,CEF
SP20	urine	1	4	1	8	16	16	PEN,AMP,DOX
SP21	urine	1	4	1	4	8	8	-
SP22	bladder swab	1	4	1	8	16	8	-
SP23	wound	1	4	1	4	8	8	PEN,AMP,
SP24	abscess	1	4	2	8	16	8	PEN
SP26	conjunctiva	1	4	1	4	4	16	AMK,BAC,CHL,ERM,NEO,OXY, TOB, TMP-SMX
SP27	epidermal collarettes (abdomen)	1	4	4	8	8	8	PEN,AMP,AMK,CHL,CLIN,ENR,ERM,GEN,MARB, TMP-SMX
SP30	tooth root abscess	1	4	1	4	8	8	PEN,AMK,CLIN,ERM,GEN, TMP-SMX
SP31	abscess	1	4	1	4	4	8	-
SP32	abscess	1	4	0.5	4	4	8	PEN,AMP,AMK,ERM,CLIN,GEN, TMP-SMX
SP33	conjunctiva	1	4	0.5	4	8	8	BAC,CIP,ERM,MOX,NEO,OXY, TMP-SMX
SP34	urine	2	4	2	8	8	8	-
SP35	urine	2	4	1	4	8	8	-
SP36	swab at bone plating	2	8	1	8	8	8	PEN,AMP,CLIN,ENR,ERM,MARB, TMP-SMX
SP37	urine	0.5	4	2	4	8	8	PEN and AMP
MIC_50_		1	4	1	4	8	8	
MIC_90_		2	8	2	8	16	16	

Abbreviation: PEN: penicillin, AMP, ampicillin, AMK: amikacin, CEF: cefpodxime, CLIN: clindamycin, GEN:gentamycin, CHL: chloramphenicol, ENR: enrofloxacin, MARB: marbofloxacin, ERM: erythromycin, BAC: bacitracin, NEO: neomycin, TOB: tobramycin, CIP: ciprofloxacin, OXY: Oxytetracyclin, TMP-SMX: trimethoprime sulphamethoxazole.

**Table 3:** Minimum inhibitory concentration (MIC) of peptides against methicillin resistant *Staphylococcus pseudintermedius* (MRSP) strains.

		MIC (µM)						Resistance phenotype
Isolates	Origin	RRIKA	RR	WR-12	IK8 “D isoform”	Penetratin	(KFF)_3_K	
SP3	orthopedic implant	1	4	0.5	4	8	4	CEF,ERM,CLIN,IMI,,OXA,TIC
SP5	urine	1	4	1	4	4	8	AMK,CEF,CHL,CLIN,ENR,ERM,GEN,IMI,MARB,OXA,TIC, TMP-SMX
SP8	urine	1	4	4	4	4	4	CEF,ERM,CLIN,IMI,OXA,TIC,CHL
SP9	skin	1	4	0.5	4	8	8	AMK,CEF,CHL,CLIN,ENR,ERM,GEN,IMI,MARB,OXA,TIC, TMP-SMX
SP25	urine	1	2	0.5	2	4	4	AMK,CEF,CHL,CLIN,ENR,ERM,GEN,IMI,MARB,OXA,TIC, TMP-SMX
SP28	urine	1	4	2	4	8	8	AMK, CEF,CHL,CLIN,ENR,ERM,GEN,IMI,MARB,OXA,TIC, TMP-SMX
SP38	implant	0.5	2	0.5	4	2	4	CEF,CHL,CLIN,ENR,ERM,GEN,IMI,MARB, OXA,TIC, TMP-SMX
SP39	ear	0.5	0.5	0.5	2	0.5	2	AMK, CEF,ENR,ERM,GEN,IMI,MARB,OXA,TIC
SP40	ear	2	4	2	4	8	8	CEF,ENR,ERM,CLIN,IMI,MARB,OXA,TIC
Sp41	urine	1	4	1	4	8	8	AMK, CEF,CHL,CLIN,ENR,ERM,GEN,IMI,MARB,OXA,TIC, TMP-SMX
MIC_50_		1	4	0.5	4	4	4	
MIC_90_		1	4	2	4	8	8	

Abbreviation:OXA: oxacillin, AMK: amikacin, CEF: cefpodxime, CLIN: clindamycin, GEN: gentamycin, CHL: chloramphenicol, ENR: enrofloxacin, MARB: marbofloxacin, ERM: erythromycin, BAC: bacitracin, NEO: neomycin, TOB: tobramycin, CIP: ciprofloxacin, OXY: Oxytetracyclin, TMP-SMX: trimethoprime sulphamethoxazole, TIC: ticarcillin, IMI: imipenem.

### Antibacterial assays

The broth microdilution technique was used to determine the minimum inhibitory concentrations (MIC) of peptides and antibiotics in MHB in accordance with the Clinical and Laboratory Standards Institute (CLSI) guidelines [17]. Peptides were dissolved in sterile distilled water containing 0.02% acetic acid. MIC assays were carried out with an initial bacterial inoculum of 5×10^5^ colony forming unit (CFU/ml). Peptides and antibiotics were added to Polystyrene 96 well plates (CytoOne, CC7672-7596) at desired concentrations and plates were incubated for 24 hr at 37°C. MIC was defined as the lowest concentration of peptide which inhibited the visible growth of bacteria. MIC was done in triplicates and the highest value was reported.

### Time kill assay

One strain of MRSP (SP3) was grown overnight in MHB, then diluted in fresh MHB and incubated for 2 hr until logarithmic growth phase was achieved. Then bacteria were centrifuged and washed twice with phosphate buffered saline (PBS) and adjusted to ∼5×10^5^ CFU/ml in MHB. Peptides and antibiotic (amikacin) at 5X MIC were added in triplicates to adjusted bacteria, and incubated aerobically at 37°C. Aliquots at specified time points were taken, serially diluted in PBS and plated in triplicate on MHA. CFUs were counted after incubation of plates for 24 hr at 37°C.

### Growth Kinetics

The turbidity of bacterial culture after exposure to peptides was measured as described previously [13]. Briefly, logarithmic growth of SP 3 was diluted in MHB to reach OD_600_≈0.25. One mL of diluted bacteria was added to a plastic cuvette (light path 1 cm). Peptides and antibiotics were added to cuvettes at a concentration equivalent to 5 X MIC as determined in antibacterial assays above. Cuvettes were incubated aerobically at 37°C. At specific time points, OD was measured at 600 nm wavelength by spectrophotometer. Cuvettes containing MHB without bacteria and with the same concentrations of peptide or antibiotic served as blanks. Nisin at 5 X MIC and untreated bacteria were used as positive and negative controls, respectively. The assay was repeated twice and the average was reported.

### Bacterial membrane permeability

Bacterial membrane damage was assessed by fluorescence change of propidium iodide dye as described previously [18]. Briefly, SP3 was grown in MHB to logarithmic phase of growth then diluted to OD_600_≈0.25. Propidium iodide was mixed with bacteria to a final concentration of 10 µM. Aliquots of 200 µL were then loaded into black wall 96 microplates and drugs were added at 5 X and 10 X MIC in triplicate. Fluorescence was monitored for 30 minutes at excitation and emission wavelengths of 585 and 620 nm, respectively, using a fluorescence plate reader (FLx800 model BioTek Instruments, Inc. Winooski, Vermont). The percentage of membrane permeabilization was calculated as the percent of fluorescent intensity of peptide-treated samples with respect to fluorescence intensity of propidium iodide-loaded, peptide untreated samples.

### Transmission electron microscopy

SP3 was grown to OD600≈0.2. Bacterial cells were harvested by centrifugation, resuspended in PBS, and treated with peptides at 10 X MIC for 30 minutes. RRIKA and penetratin were also tested at lower concentration 1 X MIC to study the concentration-dependent effect of peptides. After treatment with peptides, the bacterial pellets were fixed with 2.5% buffered glutaraldehyde for 1 hour. After fixation, cells were treated with 1% buffered osmium tetroxide for 1 hour, stained en bloc with 1% uranyl acetate, dehydrated in a graded series of ethanol, and embedded in white resin. The buffer used was 0.1M sodium cacodylate, pH 7.4. Ultrathin sections were prepared on Formvar-coated grids and stained with 1% uranyl acetate and lead citrate. Microscopy was performed with a Philips CM-100 microscope under standard operating conditions.

### Synergism studies

The synergistic effect between peptides and antimicrobial agents was determined by the combination assay as described previously [19]. Peptide MICs against SP3 were determined in triplicate. Two-fold serial dilutions of antimicrobials (antibiotic and peptides) were tested in the presence of a fixed concentration of peptide of concern (equal to ¼×peptide MIC, which did not inhibit the growth of bacteria alone). The fractional inhibitory concentration (FIC) index was calculated as follows: FIC of drug A = MIC of drug A in combination/MIC of drug A alone, FIC of drug B = MIC of drug B in combination/MIC of drug B alone, and FIC index = FIC of drug A+FIC of drug B. An FIC index of ≤0.5 is considered to demonstrate synergy. Additive was defined as an FIC index of 1. Antagonism was defined as an FIC index of >4.

### Cytotoxicity analysis

Peptides were assayed at different concentrations against macrophage-like cell line (J774A.1) and human keratinocyte (HaCat) to determine the potential toxic effect in vitro. Briefly, J774A.1 and HaCat cells were seeded at a density of 1.5×10^4^ per well in a tissue culture 96-well plate (CytoOne, CC7682-7596) in DMEM media supplemented with 10% fetal bovine serum (FBS), and incubated at 37°C in a 5% CO_2_ atmosphere for 24 hours. The cells were treated with peptides at concentrations range from 8 to 256 µM for 24 hours. Untreated cells were used as a negative control. Melittin 5 µM and DMSO 10% were used as a positive control for J774A.1 and HaCat cells, respectively. After incubation, the cells were washed three times with PBS and the media in each well were replaced with 100 µL of DMEM media and 20 µL of assay reagent, MTS 3-(4,5-dimethylthiazol-2-yl)-5-(3-carboxymethoxyphenyl)-2-(4-sulfophenyl)-2*H*-tetrazolium) (Promega, Madison, WI, USA). The cells were incubated further for 4 h at 37°C. Corrected absorbance readings (actual absorbance readings for each treatment subtracted from background absorbance) were taken using a kinetic ELISA microplate reader (Molecular Devices, Sunnyvale, CA, USA). Cell viability was expressed as percentage of absorbance in comparison to negative control, untreated cells. Therapeutic index of the peptides, was calculated as the ratio of mammalian IC_50_ (the concentration of peptide which inhibit 50% of mammalian cells) to geometric mean of the MIC of peptides against *S. pseudintermedius* [20]

### Multi-step resistance selection

To assess the ability of *S. pseudintermedius* to develop resistance to the peptides and antibiotics we used amikacin and ciprofloxacin-susceptible strain (SP6) in a multi-step resistance selection experiment (Table 2). SP6 strain was exposed for ten passages over a period of ten days to various concentrations of the peptides (RRIKA and IK8 “D isoform”) and antibiotics (amikacin and ciprofloxacin). For each subsequent passage, the inoculum for the MIC determination was adjusted to a final density of approximately 5×10^5^ CFU/mL using the bacterial cells growing at sub-inhibitory concentration (^1^/_2_ X MIC). Bacteria were incubated for 16 hr and the MIC value was recorded for each passage. Results were expressed as the ratio of MIC value after each passage compared to the initial MIC value.

### Statistical analyses

Cytotoxicity data were subjected to statistical analysis using GraphPad Prism 6.0 (GraphPad Software, La Jolla, CA). Cytotoxicity data were analyzed using one-way ANOVA, with post hoc Tukey's multiple comparisons test. *P*-values of <0.01 were considered significant.

## Results

### Antimicrobial activity

The amino acid sequences and characteristics of the synthetic peptides used in this study are shown in Table 1. All synthetic peptides demonstrated potent inhibitory activity. As shown in Table 2, the most effective peptides were RRIKA and WR-12 with MIC_50_ and MIC_90_ (the MICs at which 50% and 90% of the isolates were inhibited, respectively) were found to be 1 µM and 2 µM, respectively. RR and IK8 “D isoform” demonstrated good antimicrobial activity with MIC_50_ of 4 µM and MIC_90_ of 8 µM. Penetratin and (KFF)_3_K were the least effective with MIC_50_ of 8 µM and MIC_90_ of 16 µM.

The MICs for MRSP were not significantly different from those for the nonresistant strains. Interestingly, some peptides (such as Penetratin and (KFF)_3_K) demonstrated lower MICs values for methicillin-resistant (MIC_50_ and MIC_90_ equal to 4 and 8 µM, respectively) compared to methicillin sensitive strains (Table 3).

### Time kill assay

**Figure 1:**
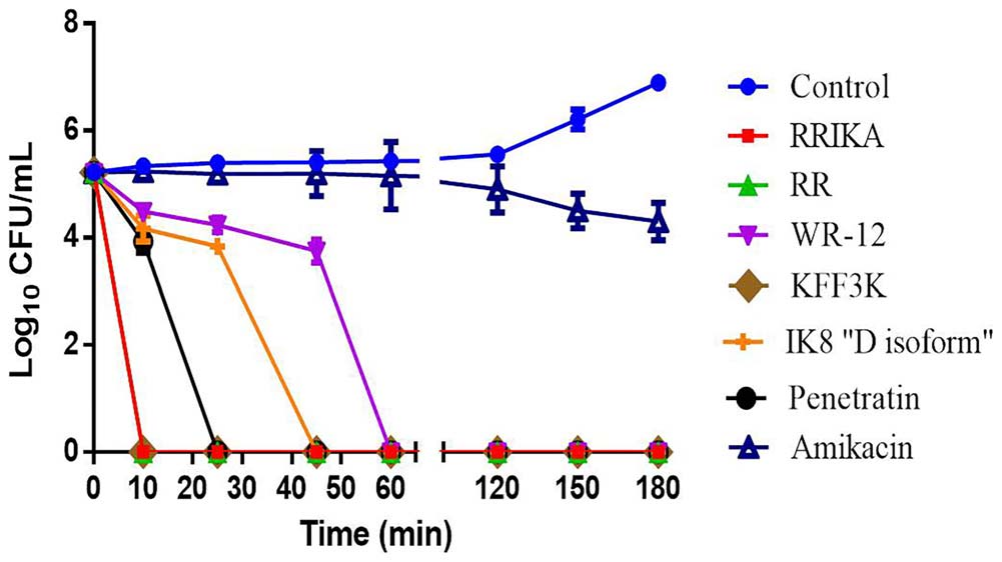
Bacterial killing kinetics of peptides (RRIKA, RR, WR-12, (KFF)_3_K, IK8 “D isoform” and penetratin) and antibiotics amikacin at 5X MIC against SP 3 in MHB (Mueller Hinton broth). Samples treated with peptide diluent (sterile water plus 0.2% acetic acid) were used as a control. The killing curves were identical (overlapping in the figure) for RRIKA, RR and (KFF)_3_K. The results are given as means ± SD (n = 3; data without error bars indicate that the SD is too small to be seen).

To determine the killing kinetics of peptides, we exposed a MRSP strain (SP3) to 5 X MIC of peptides and control antibiotic (amikacin). As depicted in Fig. 1, all peptides demonstrated rapid bactericidal activity. RRIKA, RR and (KFF)_3_K were the most rapid in killing as viable MRSP were not detectable after 10 minutes. Penetratin, IK8 “D isoform” and WR-12 achieved more than 5 log_10_ reduction within 25, 45 and 60 minutes respectively. In contrast, amikacin achieved less than one log_10_ reduction within 3 hours and demonstrated bactericidal activity only after 12 hr (data not shown).

### Growth kinetics

**Figure 2:**
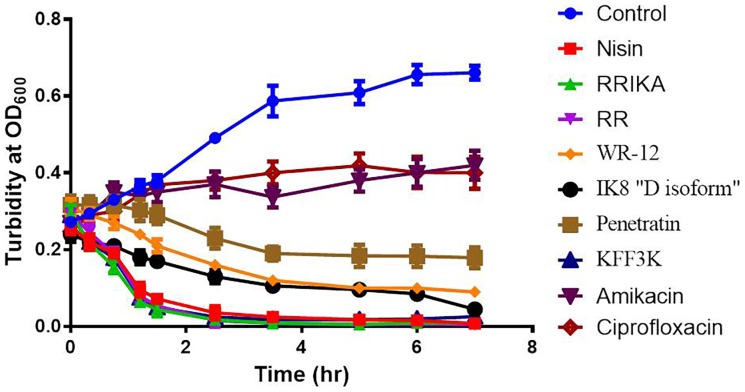
Growth kinetics of SP3 exposed to 5X MIC of peptides, antibiotics and sterile water (control) as measured by OD_600_ by spectrophotometer over time. Results are representative of two separate experiments, each done in triplicate. Error bars represent standard deviation values.

As a primary objective to understand the mechanism of action of peptides against *S. pseudintermedius*, we monitored the turbidity of SP3 culture exposed to 5 X MIC of peptides over time by a spectrophotometer. As shown in Fig. 2, all peptides reduced the optical density of *S. pseudintermedius* suspensions, and the degree of reduction over time varied among peptides. Results for RRIKA, RR and (KFF)_3_K were similar in rate and percentage to the rapidly acting membrane-damaging peptide, nisin [21]. These peptides produced greater than 50% and 95% reductions in turbidity after 60 and 120 minutes, respectively. IK8 “D isoform” and WR-12 achieved a similar percentage of turbidity reduction as nisin albeit at a slower rate (after 3 and 7 hours). However, the culture turbidity of SP exposed to penetratin remained constant for 2 hours, and turbidity was reduced by less than 50% after 7 hours. In contrast, a DNA synthesis inhibitor, ciprofloxacin, and a protein synthesis inhibitor, amikacin, did not reduce the culture turbidity within the same time frame, indicating a different mechanism of action.

### Membrane permeability

**Figure 3:**
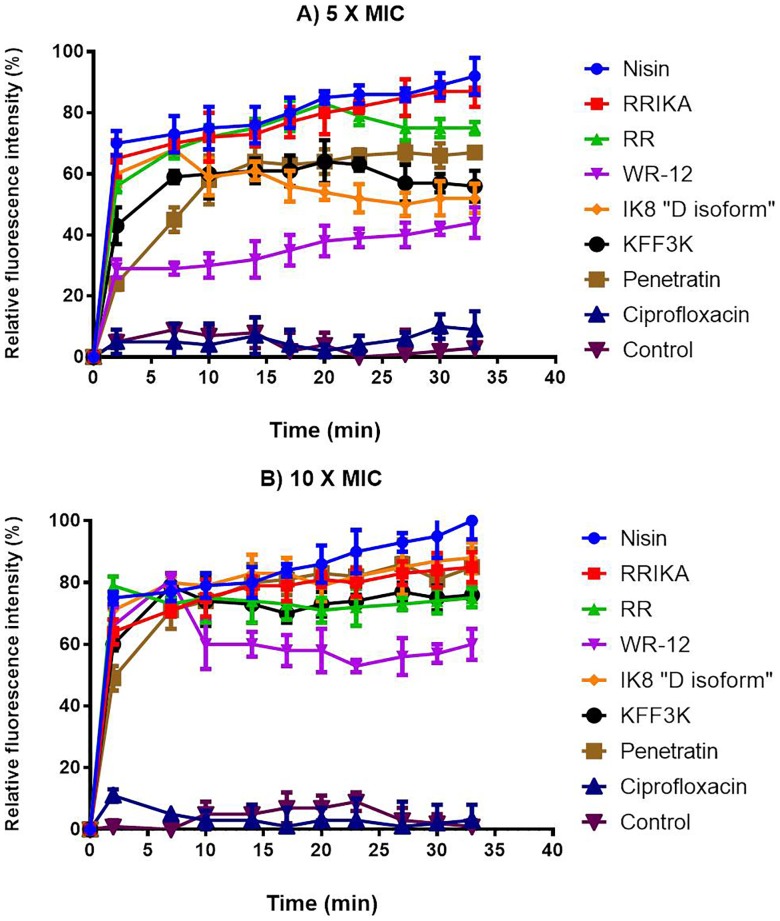
Permeabilization of the cytoplasmic membrane of SP3 as a function of peptide concentration at 5X MIC (A) and 10 X MIC (B), indicated by percent of propidium iodide fluorescence. Each experiment was done in triplicate, and the values represent means ± standard deviations.

In order to observe the permeability effect of peptides on MRSP membrane, we monitored the fluorescent intensity of bacterial culture mixed with propidium iodide (PI) after exposure to peptides. As shown in Fig. 3, fluorescence intensity remained stable during incubation of untreated bacteria in the presence of PI. This result confirmed the integrity of the bacterial membrane under control conditions. When exposed to peptides, the cells became permeable to PI and fluorescence increased within 2 minutes, suggesting very rapid membrane disruption activity. The permeabilization effect was concentration and time-dependent. At 5X MIC; nisin, RRIKA, RR and IK8 “D isoform” were faster and more potent at membrane perturbation in comparison to (KFF)_3_K, WR-12 and penetratin (Fig. 3A). However, when the concentration of peptides was increased to 10 X MIC, similar pattern was observed for all peptides, suggesting dose dependent activity (Fig. 3B). In contrast, ciprofloxacin, as expected, had no effect on membrane permeabilization, even at a higher concentration.

### Transmission electron microscopy

**Figure 4:**
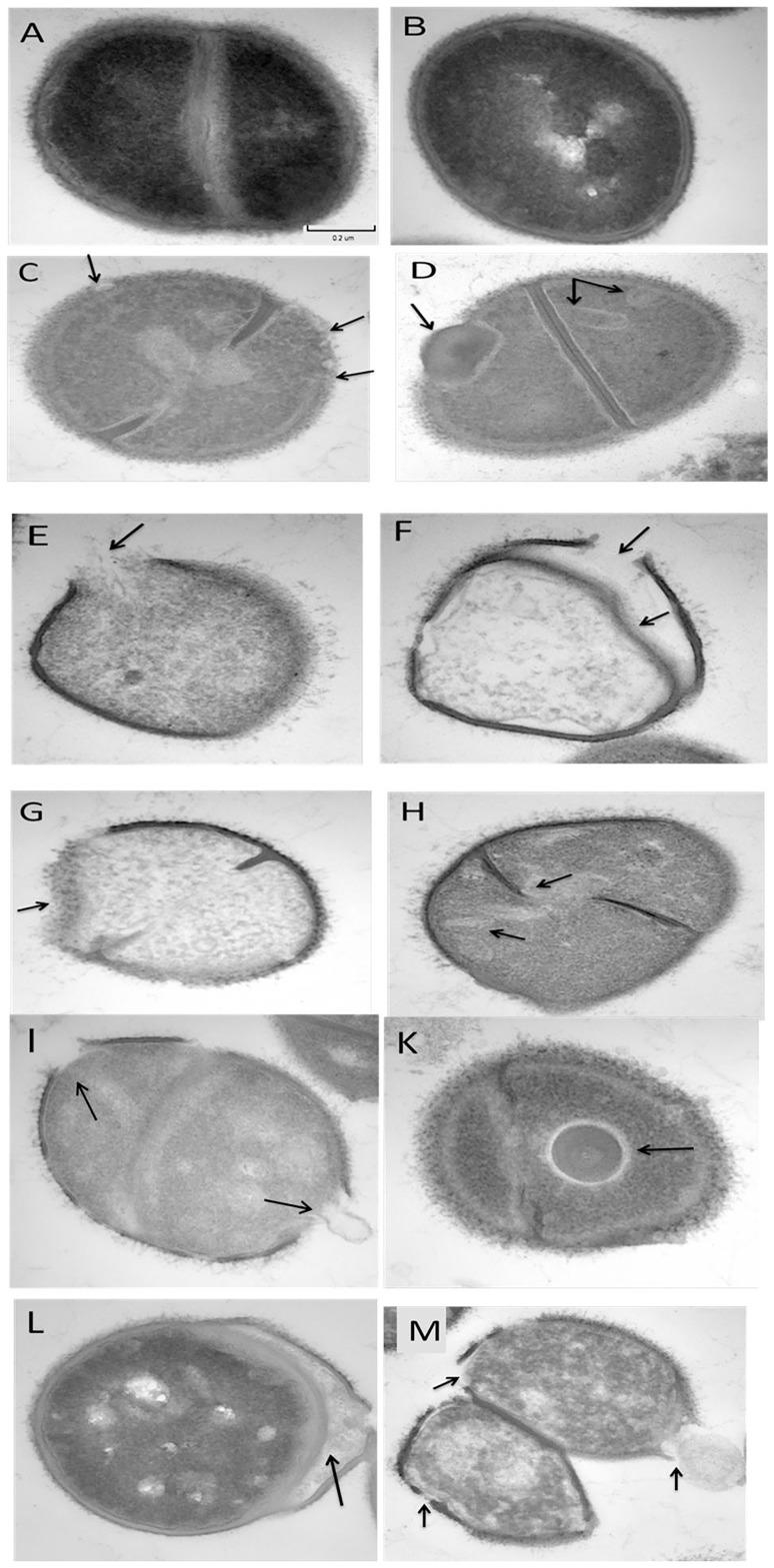
Transmission electron microscopy (TEM) micrographs of untreated and peptide treated *S. pseudintermedius* (SP3). Control bacteria either dividing cells (A) or single celled (B), the cells are round and intact, with a well-defined cell membrane. The intracellular DNA region exhibits a highly inhomogeneous electron density. C&D: 1X MIC RRIKA; E&F: 10X MIC RRIKA, G: 10X MIC RR; H: 10X MIC (KFF)_3_K; I: 10X MIC IK8 “D isoform”; K: 10X MIC WR-12; L: 1X MIC penetratin; M: 10X MIC penetratin.

To explore the possible alternative mechanism of action of peptides on *S. pseudintermedius*, transmission electron microscopy (TEM) was done on thin sections of MRSP (SP3) that had been exposed to peptides for 30 min (Fig. 4). TEM micrographs of control *S. pseudintermedius* in dividing cells (Fig. 4A) and single cells (Fig. 4B) displayed round cocci with intact cell membranes and well-defined cell wall. The nucleoid region showed a non-homogenous electron density. However, after exposure to peptides, the cytoplasm showed a more homogenous electron density (Figs. 4C, D and E). At a 1X MIC of RRIKA, multiple rounded double-layered mesosome-like structures were noticed as well as rounded bodies in the cytoplasm with similar electron density as that of the septal cell wall layer (Figs. 4C and D). At 10 X MIC of RRIKA, pore formation and lysed cells were observed (Fig. 4E&F). RR (at 10 X MIC) showed the most damaging effect on the cell wall, including alteration in cell wall thickness and cell wall rupture (Fig. 4G). (KFF)_3_K caused deviated septa and cytoplasmic inclusions. (Fig. 4H). Cell wall disintegration and pore formation could be seen in IK8 “D isoform” treated bacteria (Fig. 4I). Furthermore, some large non-membrane-enclosed bodies with similar electron density to that of the cell wall murein layer were seen in *S. pseudintermedius* cells at a 10 X MIC of WR-12 (Fig. 4K). Penetratin at 1 X MIC showed the lowest damaging effect (or didn't show clear damaging effect) as most cells had normal appearance (data not shown) with the exception of small percentage of cells that displayed a disintegrated membrane separated from the cell wall (Fig. 4L). However, at 10 X MIC, large pores were seen (Fig. 4M). Taken together, these data demonstrate the membrane-damaging effect of the investigated peptides.

### Synergism studies

As shown in Table 4, all peptides displayed synergistic interactions when combined together. The FIC index varied from 0.37 to 0.5. When peptides were combined with antibiotics; only (KFF)_3_K displayed potent synergism with amikacin and gentamicin with FIC index equal to 0.37 and 0.26, respectively. However, RRIKA, RR, WR-12, IK-8 and penetratin showed an additive effect with amikacin and gentamicin. Notably, there were no antagonistic effects observed between peptides and antibiotics.

**Table 4:** The values of FIC index for the combination of peptides and antimicrobial compounds against *Staphylococcus pseudintermedius* (SP3).

Compound	FIC index^a^					
	RRIKA	RR	WR-12	IK8 “D isoform”	Penetratin	(KFF)_3_K
RRIKA	-	0.5	0.5	0.5	0.37	0.37
RR	0.5	-	0.5	0.5	0.5	0.37
WR-12	0.37	0.5	-	0.5	0.5	0.5
IK8 “D isoform”	0.5	0.5	0.5	-	0.75	0.5
Penetratin	0.5	0.37	0.5	0.5	-	0.37
(KFF)_3_K	0.37	0.37	0.5	0.5	0.37	-
Amikacin	1	0.75	1	0.75	0.75	0.37
Gentamicin	1	0.75	0.75	1	1	0.26

a The FIC index was determined in the presence of a constant amount of peptide, equal to one -quarter of the peptide MIC.

### Cytotoxicity studies

**Figure 5:**
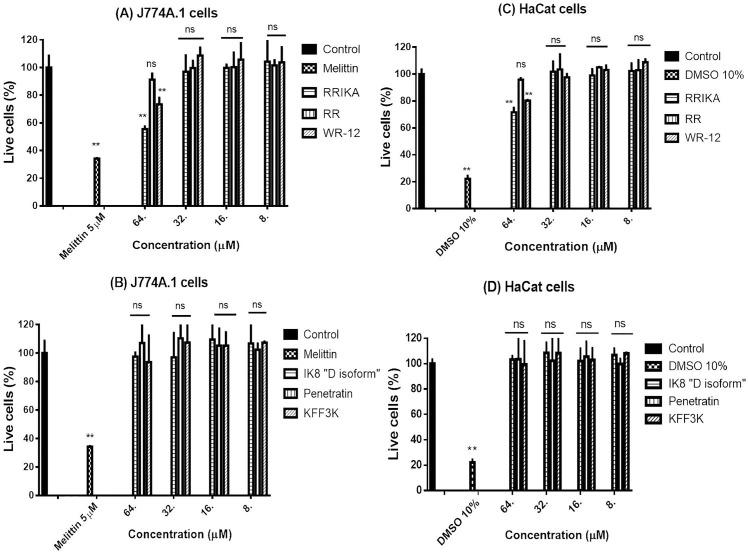
Cytotoxicity assay showing the percent mean absorbance at 490 nm after incubating macrophage cell line (J774A.1) (A &B) and human keratinocyte (HaCat) (C&D) with peptides at different concentrations. Melittin and DMSO served as positive control in J774A.1and HaCat cells, respectively. Water (peptide diluent) served as negative control. Cell viability was measured by MTS assay. Results are expressed as means from three measurements ± standard deviation. Data were analyzed using one-way ANOVA, with post hoc Tukey's multiple comparisons test to determine statistical significance. Two asterisks (**) means statistical different (p<0.01) from the negative control while (ns) means there was no statistical significance from the negative control.

To evaluate the safety of the peptides toward mammalian cells, we assessed the cytotoxic effect of different concentration of peptides on macrophage-like cell line (J774A.1) and human keratinocyte (HaCat) by the MTS assay. IK8 “D isoform” and penetratin peptides did not show any toxicity to mammalian cells at concentrations up to 256 µM. RR and (KFF)_3_K peptides were not toxic to mammalian cells at concentrations up to 64 µM (See Fig. 5). RRIKA and WR-12, were not toxic up to 32 µM, however they exhibited toxicity at 64 µM (*P*<0.01) (See Fig. 5). In contrast, melittin peptide was toxic to J774A.1at 5 µM (*P*<0.01); this concentration is equal to the MIC of the melittin.

Table 5 shows IC50 and therapeutic indices of the peptides. All peptides displayed high therapeutic index, indicating a selective action of these peptides against *S. pseudintermedius*.

**Table 5:** Cytotoxicity and therapeutic index of peptides.

Peptide	GM MIC^a^ (µM) *S. pseudintermedius*	IC_50_ b (µM) J774A.1/HaCat	TI^c^ J774A.1/HaCat
RRIKA	1.15	64/128	55.6/111.2
RR	4.12	128/256	31/62
WR-12	1.5	128/128	85.3/85.3
IK8 “D isoform”	5	>256/>256	>51.2/>51.2
Penetratin	8.76	>256/>256	>29.2/>29.2
(KFF)_3_K	7.75	256/>256	33/>33
Melittin	2	2/ND	1/ND

a GM, geometric mean of the MICs of the peptides against *S. pseudintermedius* strains.

b IC_50_ (Inhibitory Concentration 50), peptide concentration that inhibits 50% of macrophage cell line (J774A.1) or human keratinocyte (HaCat).

c TI (Therapeutic index), the ratio of the IC_50_ over the geometric mean MIC value.

ND: not determined.

### Multi-step resistance selection

**Figure 6:**
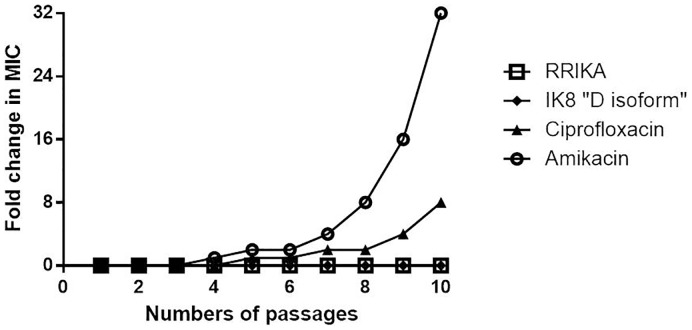
Drug resistance development profiles of *S. pseudintermedius* (SP6) exposed to ½ X MIC concentrations of peptides (IK8 “D isoform” and RRIKA) and antibiotics (amikacin and ciprofloxacin) for 10 serial passages.

In order to investigate if the physical perturbation and disruption of microbial membranes by peptides can adequately overcome conventional mechanisms of drug resistance, SP6 isolate exposed to sub-inhibitory concentrations of IK8 “D isoform” and RRIKA were subcultured for ten serial passages to determine if a shift in the MIC of each peptide tested would be observed against MSSP. Two classes of clinically used antibiotics including the protein synthesis inhibitor, amikacin and DNA synthesis inhibitor, ciprofloxacin were included as controls in this study. As shown in Fig. 6, MSSP was not able to develop resistance to the peptides despite the repeated exposure with sub-lethal doses of peptides. However, treatment of SP6 with amikacin induced a shift in the MIC as early as passage 4. There was a 32 fold increase in the MIC against amikacin by passage 10. Ciprofloxacin mediated a slightly delayed onset of drug resistance, with doubling of MIC occurring later at passage 6 which subsequently increased to 8-fold the original MIC value by passage 10.

## Discussion

The rapid emergence of bacterial resistance among *S. pseudintermedius* isolates and the dearth of effective antimicrobials call for alternative strategies to battle infections caused by this pathogen. One approach which warrants attention as a potential novel therapy for *S. pseudintermedius* infections is antimicrobial peptides (AMPs). There are very few reports describing the antimicrobial activity of naturally-derived AMPs against *S. pseudintermedius* [14], [15]. Additionally, there are several drawbacks of naturally-derived AMPs, including high toxicity and increased cost of production due to the complexity of their design and their large size, which will limit their translational clinical applications (3, 4). Given the significant problem posed by the rapid emergence of multidrug-resistant *S. pseudintermedius* and the potential benefits of using AMPs as an alternative therapy, more effort and attention needs to be focused on developing potent, short, and less toxic AMPs to overcome their therapeutic limitations. In the present study, six synthetic peptides were investigated for their ability to inhibit clinical isolates of MSSP and MRSP (see Table 1). These peptides were chosen due to their short sequence (8–16 amino acids), their simple composition, and their cationic charge. The AMPs, RR and RRIKA, are derivatives of an anti-inflammatory cell-penetrating peptide [13], [22], [23]. Previously, the efficacy of these two peptides against human MRSA clinical isolates were shown *in vitro* and in a mouse model of MRSA skin infection [13], [23]. In light of our previous success in targeting MRSA [13], [23], we tested these two peptides against MSSP and MRSP. RR exhibited potent antimicrobial activity against clinical isolates of *S. pseudintermedius* with MIC_50_ and MIC_90_ of 4 µM and 8 µM, respectively. Furthermore, addition of three amino acids (I, K and A) in the C terminus of RR significantly enhanced the antimicrobial activity fourfold against *S. pseudintermedius*. This enhancement of activity is probably due to the enhanced amphipathicity, hydrophobicity, and net charge of the newly derived peptide RRIKA. RRIKA exhibited antimicrobial activity against clinical isolates of *S. pseudintermedius* with MIC_50_ and MIC_90_ of 1 µM and 2 µM, respectively. Additionally, further advantage of using these two peptides has been demonstrated by their potent activity in physiological concentrations of NaCl and MgCl_2_, in contrast to most natural AMPs [13], [24].

Previous studies demonstrated the activity of WR-12 and IK8 “D isoform” peptides against *S. aureus* [25], [26]. However, their activity against *S. pseudintermedius* was not investigated. WR-12 is a broad-spectrum AMP and consists exclusively of arginine and tryptophan, arranged to form idealized amphipathic helices [25]. In our study, WR-12 demonstrated potent antimicrobial activity against MSSP and MRSP with MIC_50_ and MIC_90_ of 1 µM and 2 µM, respectively. Peptide IK8 “D isoform” in a previous study showed potent antimicrobial activities against MRSA [26]. Additionally, the D-amino acids substitution of this peptide prevented proteolytic degradation by mammalian or microbial proteases, which makes it an excellent candidate for *in vivo* applications [26]. Peptide IK8 “D isoform” in our study exhibited MIC_50_ and MIC_90_ of 4 µM and 8 µM, respectively, against MSSP and MRSP.

The antimicrobial activities of the cell-penetrating peptides (CPPs) have been widely explored due to their low toxicity and their potential clinical applications [27]–[31]. We sought to investigate the antimicrobial activity of two CPPs, penetratin and (KFF)_3_K, that share the same characteristic of AMPs, including basic amino acids composition and cationic charge. The CPPs showed antimicrobial activity against MSSP with MIC_50_ and MIC_90_ of 8 µM and 16 µM, respectively. Furthermore, they showed improved activity against MRSP with MIC_50_ and MIC_90_ of 4 µM and 8 µM, respectively.

We assessed the bacterial killing kinetics of the peptides in comparison with the aminoglycoside antibiotic, amikacin. The peptides showed total and rapid elimination of *S. pseudintermedius* within minutes, while amikacin exhibited a much slower killing kinetic with only 3 log_10_ reduction after 12 hours incubation (see Fig. 1). This fast bacterial elimination by the AMPs represents a unique advantage of the peptides over conventional antibiotics that should lead to better treatment outcomes.

Understanding the mechanism of antimicrobial activity of these AMPs on *S. pseudintermedius* is an essential step for enhancing their activity, reducing their toxicity, and furthering their development as a potential therapeutic option. Hence, we explored *S. pseudintermedius* membrane-perturbing abilities of these peptides. Growth kinetics study of *S. pseudintermedius* incubated with the peptides and control antibiotics revealed a membrane-damaging activity and a lytic mechanism of action similar to the lytic peptide nisin (see Fig. 2). The membrane-damaging activity of the peptides was further confirmed by the ability of propidium iodide (PI) to enter damaged *S. pseudintermedius* and intercalate with bacterial DNA. The fluorescence intensity of PI increased significantly after 2 minutes of addition of the peptides, clearly demonstrating their fast permeabilization characteristic (see Fig. 3). Additionally, transmission electron microscopy of *S. pseudintermedius* that had been exposed to the peptides for 30 minutes showed the presence of mesosome structure and damaged membranes (see Fig. 4). The observed membrane-damaging effect is similar to those seen with well-characterized AMPs such as defensins and bactenecins [32], [33].

The checkerboard assay was utilized to ascertain whether these peptides have potential to be combined with each other or with antibiotics against *S. pseudintermedius* [34]. Although peptides that work by the same mechanism of action are less likely to exhibit synergistic activity, our peptides were found to exhibit a synergistic relationship with each other against *S. pseudintermedius* (see Table 4). This finding suggests that, although these peptides share a common mechanism of action by damaging the bacterial membrane, they may use different pathways such as barrel-stave, carpet or toroidal-pore to permeabilize the bacterial membrane [9], [11]. Collectively, these results provide valuable insight into potential combination therapy that could reduce the dose of each peptide and minimize potential host toxicity and emergence of resistant pathogens. With the exception of (KFF)_3_K, none of the peptides showed synergistic activity with antibiotics. This result is probably due to their rapid bactericidal activity that masked the activity of antibiotics [19], [35], [36]. However, (KFF)_3_K is the only peptide that displayed potent synergism with the antibiotics, gentamicin and amikacin. That synergism could be explained by their ability to permeabilize the membrane, at low concentration, without killing the bacteria, which will enhance entrance of antibiotics inside bacterial cells.

A significant challenge facing AMPs which has limited their use in therapeutic applications is associated cytotoxicity to host tissues [37]. However, our peptides demonstrated high therapeutic profiles, and there was no toxicity observed on mammalian cells (J774A.1 and HaCat) at concentrations several times higher than their bactericidal concentrations (see Table 5 and Fig. 5).

After confirming the potent bactericidal activity of the peptides and their mechanism of action against *S. pseudintermedius*, we next turned our attention to assessing the likelihood that *S. pseudintermedius* would quickly develop resistance to these peptides. Repeated subculturing of *S. pseudintermedius*, in the presence of sub-inhibitory concentration, in a multi-step resistance selection for ten serial passages did not result in the development of bacterial resistance to the peptides. On the other hand, amikacin- and ciprofloxacin-resistant mutants of *S. pseudintermedius* readily emerged. The low frequency of emerging bacterial resistance to AMPs is attributed to the complexity of their mechanism of action and the extremely difficult changes required by the outer membrane to create a mutant, which cannot be acquired by simple bacterial mutation [10], [38].

We have successfully demonstrated the potential usage of AMPs against MSSP and MRSP clinical isolates. The AMPs were superior to antibiotics, demonstrating potent and rapid bactericidal activity against multidrug resistant *S. pseudintermedius* and low frequency of resistance. Moreover, AMPs such as RR and RRIKA [23] have been shown to possess potent anti-inflammatory properties and antibiofilm properties and to enhance wound healing. These are essential qualities for efficient treatment of skin infections caused by S. *pseudintermedius*. Taken together, the characteristics of these AMPs as antimicrobial agents may offer a significant improvement over current approaches and have a strong potential to be used for treatment of infections caused by multidrug resistant *S. pseudintermedius*.
